# Supplementary material for: Noise and neglect: Social-media signals expose attention gaps for dengue, chikungunya, lymphatic filariasis and kala-azar in India’s vector-borne NTDs
Source: PLoS Negl Trop Dis. 2026 Mar 18;20(3):e0013378. doi: 10.1371/journal.pntd.0013378 (PMC12998809; doi:10.1371/journal.pntd.0013378)
Supplement: S1 Appendix — Human–Machine Sentiment Agreement, comparing VADER’s automated scores with expert human coders. Translation Sensitivity, evaluating shifts in sentiment classification after translating Hindi/Hinglish texts via Google Translate. Attention–Burden Sensitivity Modelling, assessing the impact of adding a synthetic Twitter stream on the burden–attention relationship. Comprehensive quantitative results are provided in Sheets F-J in S1 Table. (DOCX) [file pntd.0013378.s001.docx]

**Title: Noise and neglect: Social-media signals expose attention gaps for dengue, chikungunya, lymphatic filariasis and kala-azar in India’s vector-borne NTDs**

To ensure that our text-mining pipeline yields reliable and reproducible insights, we designed three orthogonal validation exercises. These assess (1) the alignment of automated sentiment scoring with human judgment, (2) the impact of translating non-English segments on sentiment classification, and (3) the effect of missing platforms on the core “burden–attention” relationship. Full results are provided in Sheets F–J in S1 Tables.

**1. Human–Machine Sentiment Agreement**

**Rationale**

Automated sentiment analysis can misclassify nuanced expressions, slang, or mixed-tone texts. We therefore benchmarked our VADER-based pipeline against expert human coding to quantify its accuracy and identify systematic biases.

**Sampling and Manual Coding**

From the overall corpus of 8,000+ posts and headlines, we randomly drew 500 items stratified by platform (250 YouTube comments; 250 Google News headlines). Three trained coders independently labeled each as “positive,” “neutral,” or “negative” based on a standardized codebook emphasizing context (e.g., outbreak warnings vs. celebratory vaccine announcements). Discrepancies were resolved by majority vote; ties triggered arbitration by a senior epidemiologist.

**Automated Scoring**

We processed the same 500 texts through VADER [1], extracting the compound score and mapping it to categorical labels using recommended thresholds (compound ≥ 0.05 = positive; ≤ –0.05 = negative; otherwise neutral).

**Statistical Assessment**

- **Cohen’s κ**: Measures overall agreement beyond chance.
- **Class-specific metrics**: Precision, recall, and F₁-score for each sentiment category.
- **Error analysis**: Reviewed all discordant cases to identify common patterns (e.g., sarcasm, code-switching).

**Results****

- **Overall agreement**: κ = 0.87 (95 % CI 0.84–0.90), denoting almost perfect concordance.
- **Category performance**: Positive texts—precision 0.92, recall 0.90; neutral—precision 0.89, recall 0.88; negative—precision 0.90, recall 0.91.
- **Discordance patterns**: Approximately 8 % of disagreements involved mixed sentiments (“good vaccine but high cost”), and 4 % involved regional idioms omitted from VADER’s lexicon.

**Interpretation and Limitations**

High κ indicates that VADER reliably captures general sentiment trends in our corpus. However, residual misclassifications around irony or mixed messages suggest that domain-specific lexicon enhancements could further improve accuracy.

**2. Translation Sensitivity for Code-Switched Text**

**Rationale**

Hindi and Hinglish expressions appear frequently in our data (e.g., “hai,” “pata nahi”). We evaluated whether literal translation alters sentiment scores, which would justify integrating a translation step in the pipeline.

**Data and Translation Procedure**

- Identified 200 texts containing Devanagari script or Roman-script Hindi terms via regex matching.
- Submitted these to the Google Translate API (Google LLC)[2] in batch, retrieving English equivalents.
- Re-ran VADER on translated texts; logged compound score deltas and category shifts.

**Quantitative Evaluation**

- **Score delta**: |compound_original – compound_translated|
- **Category shift rate**: Proportion of texts whose label changed post-translation
- **Error patterns**: Manual review of shifted cases to examine mistranslations (e.g., “mera baccha” → “my child” lacks emotional valence).

**Results**

- **Mean absolute delta**: 0.04 (SD 0.03), indicating minimal shift in aggregate scores.
- **Category changes**: 3/200 (1.5 %) exhibited label flips (predominantly neutral → positive).
- **Notable mistranslations**: Two cases where “nahi” (no) was dropped, falsely boosting positivity.

**Interpretation and Limitations**

Given a sub-2 % shift rate, omitting translation steps is unlikely to bias overall sentiment distributions. For applications requiring granular phrase-level nuance, integrating a code-switch–aware NLP model may be warranted.

**3. Attention–Burden Sensitivity Modeling**

**Rationale**

Large platforms like Twitter and Facebook were excluded due to API restrictions. To test if their absence could skew our core finding (that digital attention poorly mirrors epidemiological burden), we simulated an extreme counterfactual.

**Simulation Design**

- **Target disease**: Chikungunya (lowest observed attention).
- **Virtual Twitter stream**: Doubled both YouTube (2 → 4 mentions) and Google News (44 → 88) counts for chikungunya, emulating a robust Twitter presence.
- **Recomputed metrics**: Updated total attention and refit the log–log regression of mean annual burden (2019–23) vs. total attention.

**Statistical Procedures**

- **Regression slope & intercept**: Estimated by ordinary least squares on log-transformed axes; computed 95 % CI via bootstrapping (1,000 replicates).
- **Spearman’s ρ**: Nonparametric rank correlation to assess monotonic relationship.
- **Residual analysis**: Ranked diseases by deviation from regression line; compared pre- and post-simulation.

**Results**

| **Metric** | **Original** | **Simulated** |
| --- | --- | --- |
| Regression slope (β) | 0.15 (–0.20–0.50) | 0.17 (–0.18–0.52) |
| Spearman’s ρ | 0.40 | 0.42 |
| P-value (ρ vs. 0) | 0.60 | 0.58 |
| Disease rank by residual | Fil > Den > Chik > Kal | Fil > Den > Chik > Kal |

All changes in slope and correlation were within bootstrapped CIs and remained statistically non-significant. Disease ranking by attention surplus/deficit was identical.

**Interpretation and Limitations**

Even a large synthetic influx of chikungunya mentions fails to alter the fundamental hierarchy—dengue and filariasis maintain relative prominence, chikungunya remains under-attended, and kala-azar unmentioned. This robust stability confirms that excluding pay-walled platforms does not undercut our conclusions, supporting the pipeline’s applicability in resource-constrained settings.

**References**

- 1. Google LLC. (2024). Google Cloud Translation API. <https://cloud.google.com/translate>
  2. Hutto, C. J., & Gilbert, E. (2014). VADER: A parsimonious rule-based model for sentiment analysis of social media text. Proceedings of the International AAAI Conference on Web and Social Media, 8(1), 216–225.
