# Supplementary material for: Noise and neglect: Social-media signals expose attention gaps for dengue, chikungunya, lymphatic filariasis and kala-azar in India’s vector-borne NTDs
Source: PLoS Negl Trop Dis. 2026 Mar 18;20(3):e0013378. doi: 10.1371/journal.pntd.0013378 (PMC12998809; doi:10.1371/journal.pntd.0013378)
Supplement: S2 Appendix — Includes the Communication Toolkit, Stakeholder Engagement Plan, Standard Operating Procedure (SOP), and Budget Framework. (DOCX) [file pntd.0013378.s002.docx]

**Title: Noise and neglect: Social-media signals expose attention gaps for dengue, chikungunya, lymphatic filariasis and kala-azar in India’s vector-borne NTDs**

1. **Communication Toolkit for India’s Digital NTD Campaign**

**Vision**

A strategic, multi-channel communication blueprint to enhance public visibility, deepen community engagement, and drive positive behavior change for neglected tropical diseases (NTDs) — dengue, chikungunya, lymphatic filariasis, and kala-azar.

**Core Principles**

- **Integrated Channel Use:** Employ diverse platforms—Google News, YouTube, WhatsApp, local radio, social media, and official websites—in a synchronized manner, using content tailored to each audience’s needs.
- **Evidence-Based Messaging:** Leverage digital surveillance insights (e.g., trending topics, sentiment spikes) to inform message timing and tone.
- **Cultural Relevance:** Ensure materials are bilingual (English, Hindi, and regional languages), visually accessible, and embedded in community norms.
- **Compliance:** Align with IT Rules 2021 for digital platform governance and remove misinformation proactively.

**Channel Matrix**

| **Channel** | **Audience** | **Content Format** | **Key Themes** | **Metrics** |
| --- | --- | --- | --- | --- |
| **Google News & Official Newsletters** | Urban population, policymakers, health officials | Press releases, infographics, outbreak bulletins | Dengue alerts, vaccine and elimination campaign updates | Article count, readership volume, sentiment positivity (target ≥ 70%) |
| **YouTube & Short-form Video** | Patients, caregivers, community groups | 2–5 min explainers, expert Q&A, self-care demos with captions | Lymphatic filariasis management, MDA reminders, user questions | Views, average watch time (>2 min), engagement metrics, comment tone |
| **WhatsApp / Telegram Broadcasts** | ASHAs, rural caregivers, NGOs | Text alerts, audio messages, localized visuals | Kala-azar symptoms, monsoon alerts, community outreach updates | Delivery & forward rates, interactive replies |
| **Local Radio & Podcasts** | Semi-literate, migratory labor audiences | 2-minute radio spots, drama skits, expert interviews | Dengue prevention, regional elimination efforts | Broadcast count, hotline calls, listener surveys |
| **Twitter/X & Mastodon** | Youth, media, academia | Live threads, dashboard shares, hashtag-led info campaigns | Outbreak data, vaccine updates, myth-busting | Impressions, retweets, hashtag engagement |
| **Facebook Community Pages** | Caregivers, NGOs | Carousel posts, invites, live discussions | MDA schedule, success stories, Kala-azar decline | Likes, shares, comments, attendance RSVPs |
| **NVBDCP Website & Blog** | Program managers, district officials | Reports, toolkits, webinar archives, GIS dashboards | Elimination status, self-care guides, research summaries | Page views, document downloads, dwell time |

**Rationale & Best Practices**

This integrated model aligns with RTI/Alliance’s toolkit [1] recommendation to use channel synergy, bilingual content, and real-time monitoring for health promotion effectiveness. It also leverages insights from PLOS NTDs emphasizing theory-driven campaign design and cultural tailoring.

1. **Stakeholder Engagement Plan — India’s NTD Digital Initiative**

**Purpose**

Enable structured collaboration between national agencies, field workers, researchers, NGOs, and tech platforms to ensure continuous, responsive, and culturally resonant digital communication.

**Engagement Structure**

| **Stakeholder** | **Role** | **Governance** | **Interaction Channels** | **Workflow** |
| --- | --- | --- | --- | --- |
| **NVBDCP (National)** | Exec oversight, content sanctioning, metrics oversight | Establish a Digital NTD Cell; quarterly review meetings, annual strategy workshops | Website portals, email bulletins | Approves toolkit execution, reviews dashboards, plans cyclic campaigns |
| **MoHFW & MeitY** | Policy anchoring, IT compliance oversight | Biannual inter-ministry audit, grievance redress framework | Inter-Min ministry committees, official circulars | Ensures IT Rules compliance and legal conformity |
| **State & District Health Teams** | Local content translation, field feedback, rumor flagging | Monthly coordination calls, district workshops | WhatsApp, radio, field visits | Customizes national templates; escalates local outbreaks |
| **ASHAs / Field Workers** | Community outreach, data validation, rumor countering | Linked through VHSC and community-based monitoring | WhatsApp, PHC forums | Daily deployment of materials, weekly community touchpoints, rumor tracking |
| **NGOs / Civil Society Advocates** | Local production, video moderation, impact surveys | MoUs with district teams, monthly synchronization | YouTube partnerships, local events | Co-designs vernacular content; supports live Q&As |
| **Digital Platforms (Google, YouTube)** | Content amplification, analytics provision | Technical MoUs; monthly performance reviews | Platform dashboards, data APIs | Elevates campaign content; shares actionable analytics |
| **Clinicians & Health Influencers** | Credibility in messaging | Onboarding sessions via NVBDCP, quarterly expert webinars | YouTube/Facebook Live, field interactions | Host Q&As, share verified protocols |
| **Research Institutes (e.g., CSIR-IGIB)** | Digital surveillance, analytic validation | MoUs, quarterly analytic briefings | Email, analytics dashboards | Supply sentiment/topic modeling; inform content adjustments |
| **Community Representatives** | Cultural validation, rumor reporting | Village consultations, monthly feedback | FGDs, WhatsApp | Pre-test messages; surface misinformation risk |
| **Tech & Analytics Vendors** | Monitoring architecture, alerting systems | IP-protected SLAs | APIs, dashboards | Deliver spike alerts; optimize detection algorithms |
| **Academic / Media Analysts** | Impact evaluation, independent audits | Annual convening, report submission | Reports, policy dialogues | Conduct mid- and post-campaign evaluation; suggest refinements |

**Governance & Coordination**

- **Digital NTD Cell** housed within NVBDCP drives strategy, partnerships, analytics, and legal compliance.
- **Steering Committee** with MoHFW, MeitY, and state reps ensures cross-functional alignment and rapid grievance resolution.
- **Operational Cadence**: Monthly operational calls, quarterly performance audits, annual strategy reviews.

**Rationale & Frameworks**

This plan draws from global development frameworks that emphasize [2] inclusive community participation and governance transparency . It also integrates India’s proven Village Health & Sanitation Committee model and digital surveillance practices to strengthen systemic tick-ins.

**Detailed SOP, Timeline, and Budget Framework—ready for submission to Indian government agencies (e.g., NVBDCP/MoHFW). These appendices complement the Communication Toolkit and Stakeholder Engagement Plan.**

**Standard Operating Procedure (SOP)**

**1. Objective**

To implement a multi-channel, data-driven digital communication campaign for NTDs across India, enhancing public engagement, supporting elimination goals, and aligning with digital policy norms.

**2. Scope**

Applies to all NVBDCP-led NTD communication activities across web, social media, broadcast, and grassroots platforms.

**3. Key Responsibilities**

- **Digital NTD Cell (NVBDCP)**
  - Author content calendars
  - Release official messages
  - Monitor performance metrics
  - Coordinate with MoHFW/MeitY
- **Tech & Analytics Vendors**
  - Deploy social listening tools
  - Generate weekly alerts and monthly reports
- **State/District Teams & Field Workers**
  - Localize messages
  - Disseminate content via ASHAs and community networks
- **NGOs & Influencers**
  - Produce regionally relevant video/audio content
  - Host online sessions and manage community groups
- **Digital Platforms (Google, YouTube)**
  - Support content amplification, flags, and analytics dashboards
- **Clinical Experts & Researchers**
  - Validate facts, interpret data, refine messaging

**4. Workflow**

| **Step** | **Activity** | **Timeline** | **Lead / Support** |
| --- | --- | --- | --- |
| **1. Planning** | Define monthly themes using analytics and program data | Month −1 (by 25th) | NTD Cell |
| **2. Content Draft** | Create script/graphic video drafts; route for review | Month −1 last week | NVBDCP + State + Partners |
| **3. Approval** | Final sign-off, legal & IT compliance check | Month −1 28th–30th | NTD Cell + MeitY nodal officer |
| **4. Publishing** | Schedule and deploy content per platform timelines | 1st Week of Month D | NTD Digital Cell |
| **5. Monitoring** | Track engagement, sentiment, keyword spikes weekly; take action | Weeks 1–4 | Analytics vendors + NTD Cell |
| **6. Feedback Loop** | Incorporate field stakeholder inputs; escalate emerging issues | Month-end review | Field district teams + NGOs |
| **7. Evaluation** | Analyze monthly performance against targets; adjust next cycle | Month-end +5 days | NTD Cell + Analytics team |
| **8. Governance** | Quarterly strategic depth review with MoHFW/MeitY; annual planning | Quarterly/Annually | Steering Committee |

**5. Quality Standards & Compliance**

- **Content Quality:** Bilingual, factual, culturally sensitive, 8pt minimum font.
- **Misinformation Protocol:** Monitor flagged content; escalate and respond within 48 hrs.
- **Platform Governance:** All messaging and grievance mechanisms comply with IT Rules 2021.
- **Digital Safety:** No identifiable personal data; all communications pre-screened.

**Annual Campaign Timeline**

Months 1–2: Setup

- Establish Digital NTD Cell

- Onboard vendors, platforms, and stakeholders

- Pilot digital monitoring tools

Months 3–4: Launch Campaign

- Theme: "Monsoon Dengue Prevention"

- Deploy Google News alerts, YouTube explainers, WhatsApp reminders

- Initial monitoring & sentiment tracking

Months 5–6: Scale to Filariasis

- Theme: "Lymphoedema Self‑Care & MDA Drives"

- Rollout national video series with clinical experts

- Launch community Q&A via platforms

Months 7–8: Rural Outreach

- Theme: "Kala-azar Awareness & Community Reporting"

- Localized content via NGOs, Radio skits, and WhatsApp

- Field feedback integration at month’s end

Months 9–10: Vaccine & Chikungunya Focus

- Theme: "Vaccine Updates & Post-Monsoon Preparedness"

- Live updates on Twitter/X, webinars, and blog briefings

- Social listening to inform message tone

Months 11–12: Year-End Review & Strategy Planning

- National-level performance review

- Stakeholder workshop for next-year strategy

- Budget planning for Year 2 cycle

This calendar fosters thematic continuity, seasonality-aligned responses, and builds momentum for NTD awareness year-round.

**Budget Framework**

A sample budget for a medium-scale campaign phase (e.g., Monsoon Dengue Prevention):

| **Category** | **Estimated Cost (₹)** | **Notes** |
| --- | --- | --- |
| Video Production (3 short explainer videos) | 2,50,000 | Inclusive of scriptwriting, filming, editing |
| Infographics & Animations | 1,00,000 | For Google News, blog, and WhatsApp |
| Paid Social Media Amplification | 1,50,000 | Boosted posts on Twitter, YouTube, Facebook |
| Field Distribution (posters / radio spots) | 75,000 | Supplies for rural PHCs and community centres |
| Platform Analytics & Alerts | 1,00,000/year | Subscription for listening tools & vendor support |
| Staff Time & Coordination | 3,00,000/year | Digital cell salaries, travel, meeting costs |
| Contingency (10%) | 95,000 | Buffer for unforeseen expenses |
| **Total** | **10,70,000** |  |

Scaled budgets can be developed based on geography, reach, and stakeholder engagement level. Monitoring and outcome reporting align with outcome-budgeting guidance advocated in the Central Budget Manual and Format 1 (GFR Rule 54) [3].

**References (Supplementary)**

1. RTI International. (2016). Health communication program toolkit. RTI Press. https://www.rti.org/publication/health-communication-program-toolkit
2. World Health Organization. (2017). Community engagement framework for quality, people-centred and resilient health services. WHO. <https://www.who.int/publications/i/item/community-engagement-framework-for-quality-people-centred-and-resilient-health-services>
3. Government of India. (2021). General Financial Rules (GFR), Rule 54. Ministry of Finance, Government of India. https://finmin.nic.in/sites/default/files/GFR.pdf
