# Supplementary material for: Noise and neglect: Social-media signals expose attention gaps for dengue, chikungunya, lymphatic filariasis and kala-azar in India’s vector-borne NTDs
Source: PLoS Negl Trop Dis. 2026 Mar 18;20(3):e0013378. doi: 10.1371/journal.pntd.0013378 (PMC12998809; doi:10.1371/journal.pntd.0013378)
Supplement: S3 Appendix — Detailed repository structure, prerequisites, and execution instructions for the full digital‑epidemiology pipeline—covering data acquisition, text preprocessing, sentiment analysis, topic modelling, attention‑burden computation, visualizations, robustness checks, and packaging. (DOCX) [file pntd.0013378.s003.docx]

**Noise and neglect: Social-media signals expose attention gaps for dengue, chikungunya, lymphatic filariasis and kala-azar in India’s vector-borne NTDs**

## Project Overview

This repository implements the full digital-epidemiology pipeline for analyzing public discourse on dengue, chikungunya, lymphatic filariasis, and kala-azar in India, as described in the accompanying manuscript. From raw data acquisition to robustness checks, each step is codified in standalone scripts for reproducibility.

## Prerequisites

- **Python 3.9** with packages: pandas, numpy, spacy, gensim, vaderSentiment, indic_trans, yt_dlp, GoogleNews, plotly, geopandas, cartopy, matplotlib, networkx
- **R 4.3** with packages: circlize, tidyverse
- Access to YouTube Data API credentials (for fallback) and Google Cloud Translation API v2 key

## Directory Structure

scripts/
├── 01_data_acquisition/
│ ├── scrape_youtube.py # Fetch YouTube comments via yt-dlp
│ └── scrape_news.py # Fetch Google News headlines via GoogleNews wrapper
├── 02_preprocessing/
│ ├── clean_text.py # Regex cleaning, lowercasing, emoji removal, transliteration
│ └── remove_duplicates.py # Deduplicate by SHA-256 of cleaned text
├── 03_sentiment_analysis/
│ └── vader_analysis.py # Compute VADER scores; translate Hindi fragments
├── 04_topic_modeling/
│ └── lda_topic_modeling.py # Fit LDA models for YouTube & News corpora
├── 05_attention_burden/
│ └── attention_burden.py # Normalize mentions; compute Spearman correlation; bubble map prep
├── 06_visualizations/
│ ├── plots_sankey.py # Sankey/alluvial diagrams (Plotly)
│ ├── plots_bubble_map.py # Geospatial bubble maps (GeoPandas + Cartopy)
│ └── plots_wordcloud.py # Word clouds (Python wordcloud library)
└── 07_robustness_checks/
 ├── human_machine_validation.R # Inter-rater reliability in R
 ├── translation_bias.R # Sentiment drift pre/post-translation
 └── data_exclusion_scenario.py # Scenario analysis for missing platforms

## High-Level Workflow

**Note:** This diagram illustrates the seven core phases of the analysis pipeline; the full set of 44 Python scripts and 3 R scripts are organized under these phases as detailed above in the Directory Structure.

flowchart TB
 subgraph "1. Data Acquisition"
 direction TB
 A1[scripts/01_data_acquisition/scrape_youtube.py]
 A2[scripts/01_data_acquisition/scrape_news.py]
 end
 subgraph "2. Text Pre-processing"
 direction TB
 B1[scripts/02_preprocessing/clean_text.py]
 B2[scripts/02_preprocessing/remove_duplicates.py]
 end
 subgraph "3. Sentiment Analysis"
 direction TB
 C1[scripts/03_sentiment_analysis/vader_analysis.py]
 end
 subgraph "4. Topic Modeling"
 direction TB
 D1[scripts/04_topic_modeling/lda_topic_modeling.py]
 end
 subgraph "5. Attention & Burden Comparison"
 direction TB
 E1[scripts/05_attention_burden/attention_burden.py]
 end
 subgraph "6. Visualizations"
 direction TB
 F1[scripts/06_visualizations/plots_sankey.py]
 F2[scripts/06_visualizations/plots_bubble_map.py]
 F3[scripts/06_visualizations/plots_wordcloud.py]
 end
 subgraph "7. Robustness Checks"
 direction TB
 G1[scripts/07_robustness_checks/human_machine_validation.R]
 G2[scripts/07_robustness_checks/translation_bias.R]
 G3[scripts/07_robustness_checks/data_exclusion_scenario.py]
 end

 %% Define flow between phases
 A1 --> B1
 A2 --> B1
 B1 --> B2
 B2 --> C1
 C1 --> E1
 B2 --> D1
 D1 --> E1
 E1 --> F1
 E1 --> F2
 E1 --> F3
 F1 --> G1
 F2 --> G1
 F3 --> G1
 G1 --> G2
 G2 --> G3

## Execution Instructions

1. **Install dependencies** via pip install -r requirements.txt and install.packages() in R.
2. **Run scripts in numeric order**:

- python scripts/01_data_acquisition/scrape_youtube.py
  python scripts/01_data_acquisition/scrape_news.py
  python scripts/02_preprocessing/clean_text.py
  python scripts/02_preprocessing/remove_duplicates.py
  python scripts/03_sentiment_analysis/vader_analysis.py
  python scripts/04_topic_modeling/lda_topic_modeling.py
  python scripts/05_attention_burden/attention_burden.py
  python scripts/06_visualizations/plots_sankey.py
  python scripts/06_visualizations/plots_bubble_map.py
  python scripts/06_visualizations/plots_wordcloud.py
  Rscript scripts/07_robustness_checks/human_machine_validation.R
  Rscript scripts/07_robustness_checks/translation_bias.R
  python scripts/07_robustness_checks/data_exclusion_scenario.py

1. **Outputs**:
   - Cleaned corpora: data/cleaned_youtube.csv, data/cleaned_news.csv
   - Sentiment scores: results/sentiment_summary.csv
   - Topic assignments: results/topic_distribution.csv
   - Attention–burden data: results/attention_burden.csv
   - Figures: PNGs in figures/
   - Robustness logs and metrics in robustness/

## Consolidated Master Pipeline

To streamline execution, you can use the provided pipeline.py (or notebook) that sequentially orchestrates each phase. Below is a simplified outline of pipeline.py:

#!/usr/bin/env python3
import subprocess
import sys

def run_command(cmd):
 print(f"Running: {cmd}")
 result = subprocess.run(cmd, shell=True)
 if result.returncode != 0:
 sys.exit(f"Error executing: {cmd}")

if __name__ == '__main__':
 # 1. Data Acquisition
 run_command("python scripts/01_data_acquisition/scrape_youtube.py")
 run_command("python scripts/01_data_acquisition/scrape_news.py")

 # 2. Text Pre-processing
 run_command("python scripts/02_preprocessing/clean_text.py")
 run_command("python scripts/02_preprocessing/remove_duplicates.py")

 # 3. Sentiment Analysis
 run_command("python scripts/03_sentiment_analysis/vader_analysis.py")

 # 4. Topic Modeling
 run_command("python scripts/04_topic_modeling/lda_topic_modeling.py")

 # 5. Attention & Burden Comparison
 run_command("python scripts/05_attention_burden/attention_burden.py")

 # 6. Visualizations
 run_command("python scripts/06_visualizations/plots_sankey.py")
 run_command("python scripts/06_visualizations/plots_bubble_map.py")
 run_command("python scripts/06_visualizations/plots_wordcloud.py")

 # 7. Robustness Checks
 run_command("Rscript scripts/07_robustness_checks/human_machine_validation.R")
 run_command("Rscript scripts/07_robustness_checks/translation_bias.R")
 run_command("python scripts/07_robustness_checks/data_exclusion_scenario.py")

 print("Pipeline completed successfully.")

### Usage

python pipeline.py

Alternatively, the Jupyter notebook pipeline.ipynb demonstrates each step with inline documentation and visual outputs for interactive exploration.

## Packaging as a Python Package

To distribute this pipeline as a reusable Python package we have followed these steps

**Restructure into a module**

- - Create a top-level package directory, e.g. ntd_digital_surveillance/, and move your scripts into submodules (acquisition, preprocessing, etc.) with __init__.py files.
  - Expose key functions (e.g., scrape_youtube(), clean_text(), run_pipeline()) in the package’s public API.

1. **Add packaging metadata**
   - Include a pyproject.toml or setup.py with fields: name, version, author, description, url, license, packages, install_requires, and entry_points for console scripts (ntd-survey = ntd_digital_surveillance.cli:main).
   - Provide a LICENSE (e.g., MIT or Apache 2.0) and README.md for PyPI.
2. **Documentation & Tests**
   - Write API docs using Sphinx or MkDocs, host on Read the Docs.
   - Add unit tests in tests/, covering each module with pytest, and configure continuous integration (GitHub Actions).
3. **Versioning & Citation**
   - Follow semantic versioning (MAJOR.MINOR.PATCH).
   - Include a CITATION.cff file to enable JOSS and others to cite your software.
4. **Publishing**
   - Register and upload releases to PyPI with twine.
   - For JOSS submission, prepare a short paper (abstract, statement of need, references) and link the repository, documentation, and CITATION file.

By packaging the scripts into a well-documented, test-covered library with a proper CLI and metadata, a sustainable tool has been created that users can install via pip install ntd-digital-surveillance and cite in scholarly venues.

## Snakemake Workflow

To orchestrate the entire pipeline with Snakemake, a top‑level Snakefile is created in the repository root. Below is a minimal example:

# Snakefile

# Define config and paths
define:
 RAW_DIR = 'data/raw'
 CLEAN_DIR = 'data/clean'
 RESULTS_DIR = 'results'
 FIGURES_DIR = 'figures'
 SCRIPTS = 'scripts'

rule all:
 input:
 expand(f"{CLEAN_DIR}/cleaned_youtube.csv"),
 expand(f"{RESULTS_DIR}/sentiment_summary.csv"),
 expand(f"{FIGURES_DIR}/bubble_map.png"),
 # add other final outputs

rule scrape_youtube:
 output:
 f"{RAW_DIR}/youtube.json"
 shell:
 "python {SCRIPTS}/01_data_acquisition/scrape_youtube.py --output {output}"

rule scrape_news:
 input:
 f"{RAW_DIR}/youtube.json"
 output:
 f"{RAW_DIR}/news.json"
 shell:
 "python {SCRIPTS}/01_data_acquisition/scrape_news.py --output {output}"

rule clean_text:
 input:
 youtube=f"{RAW_DIR}/youtube.json",
 news=f"{RAW_DIR}/news.json"
 output:
 youtube_clean=f"{CLEAN_DIR}/cleaned_youtube.csv",
 news_clean=f"{CLEAN_DIR}/cleaned_news.csv"
 shell:
 "python {SCRIPTS}/02_preprocessing/clean_text.py --in1 {input.youtube} --in2 {input.news} --out1 {output.youtube_clean} --out2 {output.news_clean}"

# Define additional rules for deduplication, sentiment, topic_modeling, etc.
# Each rule wraps a script from the scripts/ directory, passing inputs and outputs as parameters.

Place this Snakefile at the repo root. You can then run:

snakemake --cores 4

to execute the full workflow with automatic dependency resolution.

## Docker Containerization

A Dockerfile at the root is created to encapsulate dependencies and environment:

# Use official Python image with R installed
FROM rocker/verse:4.3.0

# System dependencies
RUN apt-get update && apt-get install -y \
 libspacy-dev python3-spacy \
 libgeos-dev libproj-dev \
 && rm -rf /var/lib/apt/lists/*

# Python environment
COPY requirements.txt /tmp/
RUN pip install --no-cache-dir -r /tmp/requirements.txt

# R packages
RUN R -e "install.packages(c('circlize','tidyverse'), repos='https://cloud.r-project.org')"

# Copy repository
WORKDIR /home/project
COPY . /home/project

# Entry point for the pipeline
ENTRYPOINT ["snakemake"]
CMD ["--cores", "1"]

Build and run:

docker build -t ntd-survey-pipeline .
docker run --rm -v $(pwd):/home/project ntd-survey-pipeline --cores 4

**GitHub Release, License & DOI via Zenodo (Biswal et al., 2025)**

- We chose an open‑source license (MIT or Apache 2.0) and added a LICENSE file to the repository. We initialized the Git repository and made our initial commit:
- bash
- CopyEdit
- git init ntd-digital-surveillance
- git add .
- git commit -m "Initial commit: digital‑surveillance pipeline"
- git remote add origin https://github.com/your-org/ntd-digital-surveillance.git
- git push -u origin main
- Next, we created and tagged our first GitHub release:
- bash
- CopyEdit
- git tag -a v1.0.0 -m "First release"
- git push origin v1.0.0
- Finally, we signed into Zenodo, enabled our GitHub–Zenodo integration for the repository, and minted a DOI for the v1.0.0 release [1].

**Digital Surveillance Pipeline for India’s Vector‑Borne NTDs**
**Biswal DK, Konhar R, Lalsanga JK**

**Abstract**
We present **ntd‑digital‑surveillance**, a reproducible Snakemake‑based pipeline for the automated collection and analysis of online signals related to India’s four major vector‑borne neglected tropical diseases (NTDs): dengue, chikungunya, lymphatic filariasis, and kala‑azar. This open‐source tool integrates YouTube and Google News scraping, sentiment and keyword tracking, and Docker encapsulation to facilitate rapid digital surveillance and inform public‑health communication strategies.

**Introduction**
Digital surveillance of social and news media can highlight attention gaps and emerging outbreaks of NTDs, guiding targeted interventions. However, assembling heterogeneous data streams at scale remains a challenge. We introduce a modular pipeline that automates data ingestion, processing, and basic analytics, lowering the barrier for researchers and health agencies to monitor online discourse around NTDs.

**Methods**

- **Workflow orchestration**: Built on **Snakemake**, the pipeline defines a series of rules for data fetching, cleaning, and feature extraction.
- **Data sources**:
  1. **YouTube** — video metadata, view counts, comments
  2. **Google News** — article headlines, publication dates, source metadata.
- **Containerization**: All dependencies (Python packages, API clients) are packaged via **Docker**, ensuring reproducibility across environments [DOI](https://doi.org/10.5281/zenodo.15883324).

**Implementation & Usage**

1. Clone the GitHub repo:

bash

CopyEdit

git clone https://github.com/devbioinfo/ntd-digital-surveillance

1. Build and run the Docker image:

bash

CopyEdit

docker build -t ntd-surv:v0.1.0 .

docker run --rm -v $(pwd)/data:/data ntd-surv:v0.1.0 snakemake --cores 4

1. Outputs (CSV and JSON) are written to data/, including time‑series of mention counts and basic sentiment tags.

**Availability & License**

- **Source code & container**: <https://github.com/devbioinfo/ntd-digital-surveillance> [DOI](https://doi.org/10.5281/zenodo.15883324)
- **Zenodo DOI**: <https://doi.org/10.5281/zenodo.15883324>
- **License**: MIT (permitting reuse with attribution) [DOI](https://doi.org/10.5281/zenodo.15883324)

**Conclusion**
By automating multi‑platform data collection and formatting, this pipeline supports timely, data‑driven decision making for public‐health stakeholders addressing India’s NTD burden. Future extensions may incorporate Twitter/X and WhatsApp signal streams.

**References**

- 1. Biswal DK, Konhar R, & Lalsanga JK (2025). *Digital Surveillance Pipeline for India’s Vector‑borne NTDs* (v0.1.0). Zenodo. <https://doi.org/10.5281/zenodo.15883324>
