# Supplementary material for: Noise and neglect: Social-media signals expose attention gaps for dengue, chikungunya, lymphatic filariasis and kala-azar in India’s vector-borne NTDs
Source: PLoS Negl Trop Dis. 2026 Mar 18;20(3):e0013378. doi: 10.1371/journal.pntd.0013378 (PMC12998809; doi:10.1371/journal.pntd.0013378)
Supplement: Attachment — Submitted filename: Response to reviewers.pdf [file pntd.0013378.s007.pdf]

Response to Reviewers and Editors

Dear Dr. Loong and Editorial Team,

We thank the reviewers and editor for their thoughtful comments on our manuscript **(PNTD-D-25-01183, "Noise and neglect: Social-media signals expose attention gaps for dengue, chikungunya, lymphatic filariasis and kala-azar in India's vector-borne NTDs").** We have carefully revised the manuscript to address these points, focusing on improving figure/table quality, simplifying the presentation (especially Figure 5), reducing redundancy, and making the text more concise and accessible without losing scientific rigor. In summary, all figures and tables have been improved (higher resolution and clarity), Figure 5 has been simplified, redundant material has been removed, only essential figures/tables remain, and the Discussion has been restructured to follow the order of the stated objectives. Below, we respond to each comment point-by-point.

Manuscript ID: PNTD-D-25-01183

Title: Noise and neglect: Social-media signals expose attention gaps for dengue, chikungunya, lymphatic filariasis and kala-azar in India's vector-borne NTDs

Journal Requirements

1. Requirement: Please submit a revised version… several reviewers pointed to the need to provide better quality and simpler and clearer figures.

Response: We have regenerated all figures using high-resolution settings (300 dpi) and checked them against the PLOS NAAS tool requirements. We have specifically simplified Figure 5 (now separated into term frequency and sentiment butterfly plots) to improve readability.

2. Requirement: If the reviewer comments include a recommendation to cite specific previously published works….

Response: We have reviewed the references. We have ensured all citations are relevant and have followed the journal's formatting guidelines.

Reviewer #1 Comments

1. Comment: Authors should relook at the way the results were presented. Consider simplifying Figure 5. Also, please improve all figure quality.

Response: Thank you for this feedback. We have significantly revised the presentation of the results. Regarding Figure 5, we have completely redesigned it. Instead of a complex composite, it now clearly presents Term-Frequency distributions (Panels A and B) and a

distinct Sentiment Butterfly Plot (Panel C) to clearly separate lexical frequency from sentiment polarity. All figures have been re-exported at high resolution to ensure text and data points are crisp.

2. Comment: The manuscript's impact is reduced by its length and redundancy... I strongly encourage the authors to make the writing more concise and impactful.

Response: We agree with the reviewer. We have edited the manuscript to remove repetitive phrasing in the Results and Discussion sections. We have tightened the text to focus on the key "attention–burden mismatch" and the specific platform niches (Google News vs. YouTube).

3. Comment: The results should also be presented in a manner accessible to non-expert audiences.

Response: We have simplified the narrative, particularly in the Author Summary and Introduction, to ensure the findings are accessible to a broader audience. We have also added a "Policy Roadmap" in the Discussion to provide concrete, actionable takeaways for non-expert stakeholders.

Reviewer #2 Comments

1. Comment: Tables and figures quality must be improved. The results are quite clear, but they should be presented in a simpler and more accessible way.

Response: We have overhauled the visual elements of the paper. We used Python (Matplotlib/Seaborn) and R (Circlize) to generate clean, high-contrast figures . We have ensured that the textual descriptions in the Results section directly guide the reader through the figures to improve accessibility.

2. Comment: Please reconsider Figure 5 — simplify it and clarify how the authors are using this presentation.

Response: As noted in our response to Reviewer #1, Figure 5 has been simplified to focus on "Frequency footprints" and "Sentiment patterns" separately. We have updated the figure legend and the corresponding results text to explicitly explain how these term frequencies reflect the "linguistic shape" of the public discussion.

3. Comment: Improve all figures quality (resolution).

Response: We have ensured all figures meet the standard print requirements (TIFF/PNG at 300 dpi) and verified them using the PLOS NAAS tool as recommended.

Reviewer #3 Comments

1. Comment: The manuscript should be shortened to reduce redundancy and repetition, with a stronger focus on presenting only essential figures and tables.

Response: We have condensed the manuscript by removing overlapping text between the Results and Discussion. We have focused on the most critical visual evidence: the Waffle Charts (Fig 1), the Sankey/Chord diagrams (Fig 2), and the Network Analysis (Fig 6), ensuring every figure directly supports the core argument of the "attention gap".

2. Comment: The discussion of findings should be more closely aligned with the sequence of objectives and results to improve logical flow and reader comprehension.

Response: We have restructured the Discussion to mirror the order of our objectives. The new structure is: 1. **Digital Attention Landscape** (matching Objective 1: Quantify attention). 2. **Sentiment and Thematic Patterns** (matching Objective 2: Describe sentiment/themes). 3. **The Attention–Burden Mismatch** (matching Objective 3: Compare attention vs. burden). This creates a linear narrative that is easier to follow.

3. Comment: The numbers and the words in figures are blur and difficult to read.

Response: We apologize for the low resolution in the previous submission. We have re-generated all static graphics using vector-based or high-DPI raster outputs to ensure all text, numbers, and labels are sharp and legible at 100% zoom.

Once again, we appreciate the reviewers' and editor's comments, which have greatly improved the manuscript. We trust that the revised version meets the journal's criteria and addresses all concerns. Thank you for considering our revisions.

Sincerely,

Devendra Kumar Biswal (Corresponding author)

Ruchishree Konhar

James K. Lalsanga
