# Supplementary material for: Noise and neglect: Social-media signals expose attention gaps for dengue, chikungunya, lymphatic filariasis and kala-azar in India’s vector-borne NTDs
Source: PLoS Negl Trop Dis. 2026 Mar 18;20(3):e0013378. doi: 10.1371/journal.pntd.0013378 (PMC12998809; doi:10.1371/journal.pntd.0013378)
Supplement: Attachment — Submitted filename: RESPONSE_TO_REVIEWERS_auresp_2.pdf [file pntd.0013378.s008.pdf]

RESPONSE TO REVIEWERS

Manuscript ID: PNTD-D-25-01183R1

Title: Noise and neglect: Social-media signals expose attention gaps for dengue, chikungunya, lymphatic filariasis and kala-azar in India's vector-borne NTDs

Dear Dr. Loong, Dr. Kamhawi, Dr. Paul Paul Brindley and esteemed Reviewers,

We sincerely thank the Academic Editor, Editor-in-Chief, and all three reviewers for their thoughtful evaluation of our manuscript and for recommending acceptance pending minor revisions. We are grateful for the constructive suggestions, which have further strengthened the clarity and presentation of our work.

Below, we respond point-by-point to each comment.

Reviewer #1

We thank the reviewer for their positive assessment and for recognizing the public health relevance and novelty of our study.

Comment:

Authors made all necessary corrections based on the comments.

Response:
We sincerely appreciate the reviewer's supportive evaluation and encouraging remarks regarding the study's contribution to bridging public perceptions and public health priorities.

Comment (Ethical/Regulatory Concern marked "Yes")

Response:
We have clarified the ethical considerations in the Methods section to explicitly state that all data used in this study were publicly available, aggregated, and de-identified. No individual-level or private data were accessed. Therefore, institutional ethical approval was not required. This clarification has been added to improve transparency.

Reviewer #2

We thank the reviewer for highlighting the strengthened conceptual framing and the value of the platform-specific analysis.

Comment: Briefly reiterate that digital attention and sentiment do not equate to awareness or information quality.

Response:
We agree with this important clarification. We have added a paragraph in the Discussion emphasizing that digital engagement metrics reflect patterns of online attention and do not necessarily indicate population-level awareness, knowledge accuracy, or information quality(**570-573**).

Comment:

Reinforce the epidemiological caveat regarding lymphatic filariasis case counts when interpreting attention–burden ratios.

Response:
We appreciate this insightful suggestion. We have strengthened the Discussion to clarify that declining reported LF case counts under elimination programs may not fully capture residual morbidity or transmission dynamics. Accordingly, attention–burden ratios for LF should be interpreted cautiously within this broader epidemiological context (**Lines 276-280 and 576-582**)

Reviewer #3

We thank the reviewer for their positive evaluation of the methods and conclusions.

Comment:

Figures are very blur and difficult to read the numbers.

Response:
We have regenerated all figures in high-resolution TIFF format (≥600 dpi) in full compliance with PLOS NTDs technical and NAAS tool requirements, including optimized font size, contrast, and vector elements where applicable.

However, it appears that the Editorial Manager system preview does not display the full uploaded resolution. This limitation is related to the platform's rendering settings rather than the submitted files. To ensure reviewers can access publication-quality figures, each figure in the generated PDF contains a direct download link (top-right corner) that allows retrieval of the original high-resolution version (e.g., Figure 3 screenshot given below and the arrow point where to download). We kindly request reviewers to use these links when evaluating figure clarity.

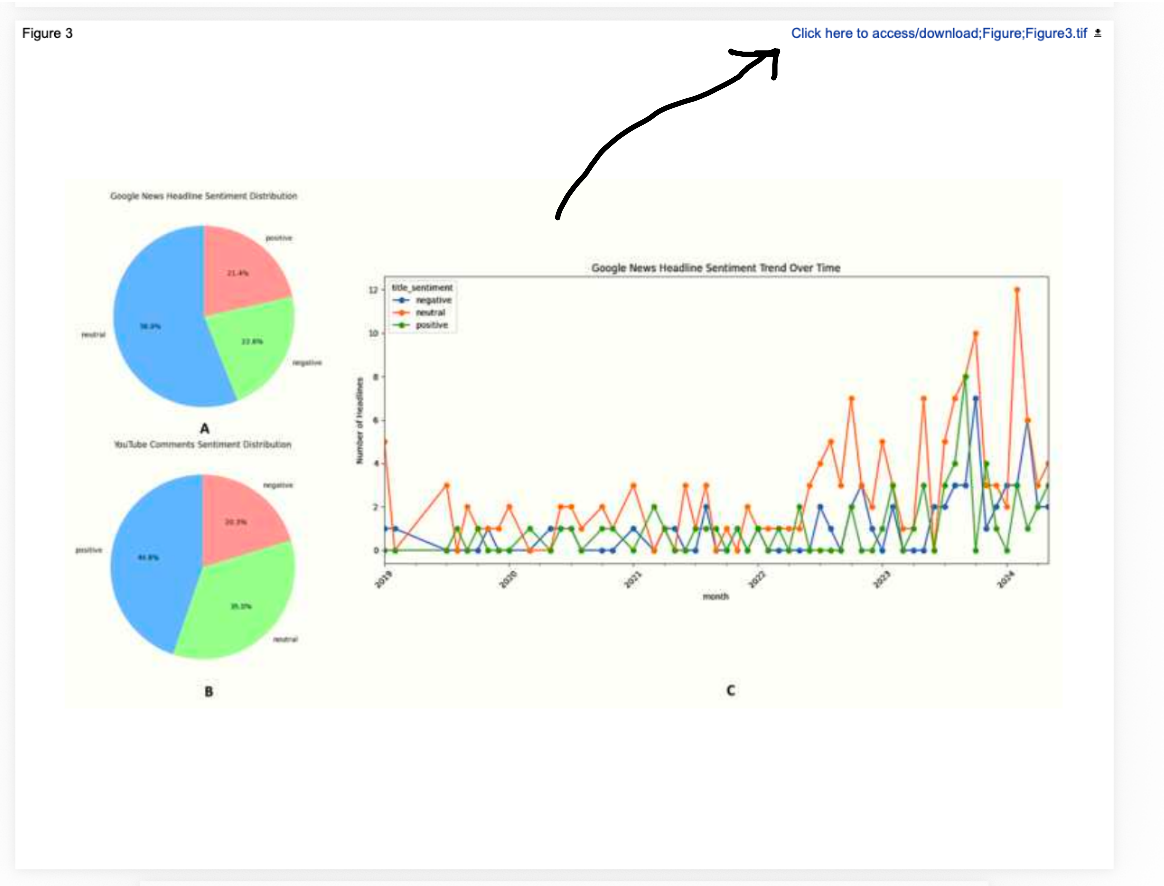

We believe the revised figures are now clear, legible, and publication-ready.

We are grateful to the editors and reviewers for their constructive guidance, which has helped us improve both clarity and presentation. We respectfully submit the revised manuscript for your consideration.

With sincere thanks,

Dr. Devendra Kumar Biswal
on behalf of all authors
